# Portable SA/CMC entrapped bimetallic magnetic fly ash zeolite spheres for heavy metals contaminated industrial effluents treatment via batch and column studies

**DOI:** 10.1038/s41598-022-07274-5

**Published:** 2022-03-02

**Authors:** Ganesh Kumar Reddy Angaru, Yu-Lim Choi, Lakshmi Prasanna Lingamdinne, Janardhan Reddy Koduru, Jae-Kyu Yang, Yoon-Young Chang, Rama Rao Karri

**Affiliations:** 1 Department of Environmental Engineering, Kwangwoon University, Seoul, 01897 Republic of Korea; 2 Petroleum and Chemical Engineering, Faculty of Engineering, Universiti Teknologi Brunei, Bandar Seri Begawan, BE 1410 Brunei Darussalam

**Keywords:** Environmental sciences, Chemistry, Materials science

## Abstract

Heavy metals are perceived as a significant environmental concern because of their toxic effect, bioaccumulation, and persistence. In this work, a novel sodium alginate (SA) and carboxymethylcellulose (CMC) entrapped with fly ash derived zeolite stabilized nano zero-valent iron and nickel (ZFN) (SA/CMC-ZFN), followed by crosslinking with CaCl_2_, is synthesized and applied for remediation of Cu(II) and Cr(VI) from industrial effluent. The characterization of the adsorbent and its surface mechanism for removing metals were investigated using advanced instrumental techniques, including XRD, FT-IR, SEM–EDX, BET, and XPS. The outcomes from the batch experiments indicated that monolayer adsorption on homogeneous surfaces (Langmuir isotherm model) was the rate-limiting step in both heavy metals sorption processes. The maximum adsorption capacity of as-prepared SA/CMC-ZFN was 63.29 and 10.15 mg/g for Cu(II) and Cr(VI), respectively. Owing to the fact that the wastewater released from industries are large and continuous, a continuous column is installed for simultaneous removal of heavy metal ions from real industrial wastewater. The outcomes revealed the potential of SA/CMC-ZFN as an efficient adsorbent. The experimental breakthrough curves fitted well with the theoretical values of Thomas and Yoon-Nelson models. Overall, the results indicated that SA/CMC-ZFN is a viable, efficient, and cost-effective water treatment both interms of batch and column processes.

## Introduction

In recent times, considerable attention has been paid to heavy metal removal from aqueous solutions. Heavy metals are harmful to humans even at low concentrations, and cause multiple illnesses and disorders after entering the human body^1^. Industrial effluents consist of greater Cr(VI) and Cu(II) levels from metal extraction, electroplating, mining, leather tanning, and paper manufacturing^2^. Cr(VI) is a highly soluble and mobile hazardous metalloid, while Cu(II) has detrimental and bioaccumulate^3^. Many techniques are used to remove heavy metals from aqueous solutions, including ion exchange, precipitation, adsorption, coagulation/flocculation, and reverse osmosis. Adsorption has proven to be the most successful method for removing heavy metals owing to its ease of use, practicability in the field, and low installation cost^4,5^. The adsorbent characteristics mostly determine the adsorption process.

Among the green adsorbents, polysaccharides are found to be more potent. These adsorbents are biodegradable and cheaper^6^. In this context, the natural polymer sodium alginate (SA) is extensively utilized as an encapsulating compound and a composite material, which may create a 3-D gel by exchanging sodium ions with divalent cations (e.g., Ca(II))^7^.

However, SA has several issues, such as lower water resistance, severe deterioration under heat, and poor stability^8^. Therefore, SA must be modified before its use in removal of heavy metals. Carboxymethyl cellulose (CMC) is an anionic polysaccharide and cellulose ether polymer, with carboxyl and hydroxyl groups^9^. In water treatment, a combination of SA and CMC has been utilized to remove heavy metal ions from wastewater^10^. However, their larger applications is hampered by lower adsorption capability for some heavy metals and a lack of stability. As a result, researchers have concentrated on creating polysaccharides/inorganic composite materials, such as graphene oxide^11^, SiO_2_^12^, and clay^7^, that can increase the stability and adsorption capability of polysaccharides-based adsorbents.

On other side, Coal fly ash (CFA) generated from the thermal power plant contains inorganic composites, that harm the environment by polluting surface water with toxic heavy metals. Therefore, promoting effective waste recycling methods that help produce high-value-added goods is essential. The synthesis of zeolites from CFA is a viable method for waste reuse^13^. Furthermore, CFA is a source of silicon and aluminium, and its cost-effectiveness has sparked interest in zeolite synthesis^14^. Zeolite has the potential to be a valuable material in wastewater treatment.

Furthermore, zeolite has demonstrated a high selectivity for different heavy metal ions^13,15^. However, the use of pristine zeolite in practical applications is disadvantageous because it exhibits poor removal capability for certain heavy metals. To overcome these issues, zeolite modification with nanoparticles is necessary.

Nano zero-valent iron (nZVI) has many salient features like non-toxicity, high activity, and cheaper, in addition to a larger surface area. nZVI has been widely utilized to remove different contaminants from aqueous solutions due to its high reducing activity^16^. Furthermore, nZVI efficiently removes various heavy metals from wastewater by reduction, adsorption, and co-precipitation^3,17^. Recent researches have shown that metallization may significantly increase the catalytic characteristics of monometallic Fe^0^. To improve the reactiveness and reduction ability of nZVI, a bimetallic composite was created by combining Fe with Pt, Pd, Cu, and Ni^18–20^. Nickel is more suited for practical application among the produced bimetallic nanoparticles due to their lower toxicity and economic feasibility^21^. However, these nanoparticles are difficult to separate from aqueous solutions, limiting their use in large-scale water treatment systems. Furthermore, when used in continuous flow streams, their leakage or leaching may create secondary pollution. To address these issues, a technique is developed for immobilizing nZVI particles by entrapment in a porous polymeric bead^22^.

In the recent studies, synthesized composites such as zeolite/PVA/SA beads^23^, core–shell nZVI@SA/CMC^10^, alginate/CMC/ZnO nanoparticles^24^, chitosan-nZVI-activated carbon^25^, etc., were used for removal of heavy metals and organic pollutants from water. The polymer matrix could enhance the distribution of particles and improve the mechanical character of polymer. Additionally, polymer encapsulation can prevent particle agglomeration, leading to high reactive sites for adsorption. Moreover, the low density of porous beads makes them floatable and thus convenient to separate. However, no artice is available in open literature on developing the fly ash derived zeolite supported nano zero-valent iron and nickel (ZFN) entrapping using polymer matrix or combination polymers and applied for water treatment. Thus, the merits of the SA/CMC polymer matrix were used for entrapping the ZFN to improve the real applicability of wastewater treatment.

In this research study, SA/CMC entrapped bimetallic magnetic fly ash zeolite spheres (SA/CMC-ZFN) were synthesized and well-characterized utilizing sophisticated instrumental techniques for the removal of Cr(VI) and Cu(II) from industrial wastewater. The batch adsorption studies were examined under various conditions, such as pH effect, kinetics and adsorption isotherms, adsorption mechanism, co-existing ions effect, and temperature effect. Furthermore, the adsorption process mechanism were analysed using spectroscopic techniques. Owing to the fact that the wastewater released from industries are large and continuous, a continuous column is installed for simultaneous removal of heavy metal ions from real industrial wastewater as well as to understand the feasibility and applicability of the developed material in the real water system.

## Materials and methods

### Chemicals

The reagent FeCl_3_.6H_2_O (97% purity), NaBH_4_ (98% purity), Carboxymethyl cellulose sodium salt, CaCl_2_ (96% purity), NaOH, and HCl were procured from “Samchun Pure Chemical Co. Ltd, Korea” and Ni(NO_3_)_2_.6H_2_O (97% purity) and Sodium Alginate were supplied by “Junsei Chemical Co., Ltd., Japan”. The commercial natural zeolite was purchased from Bear River Zeolite, a United States Antimony Corporation division. 1000 mg/L of Cu(II) and Cr(VI) solutions were prepared from Cu(NO_3_)_2_. 3H_2_O (99.5% purity) and K_2_CrO_4_ were purchased from “Duksan pure chemical co., Ltd, South Korea” and “Junsei Chemical Co., Ltd., Japan”, respectively.

### Synthesis of SA/CMC-ZFN

**Figure 1:**
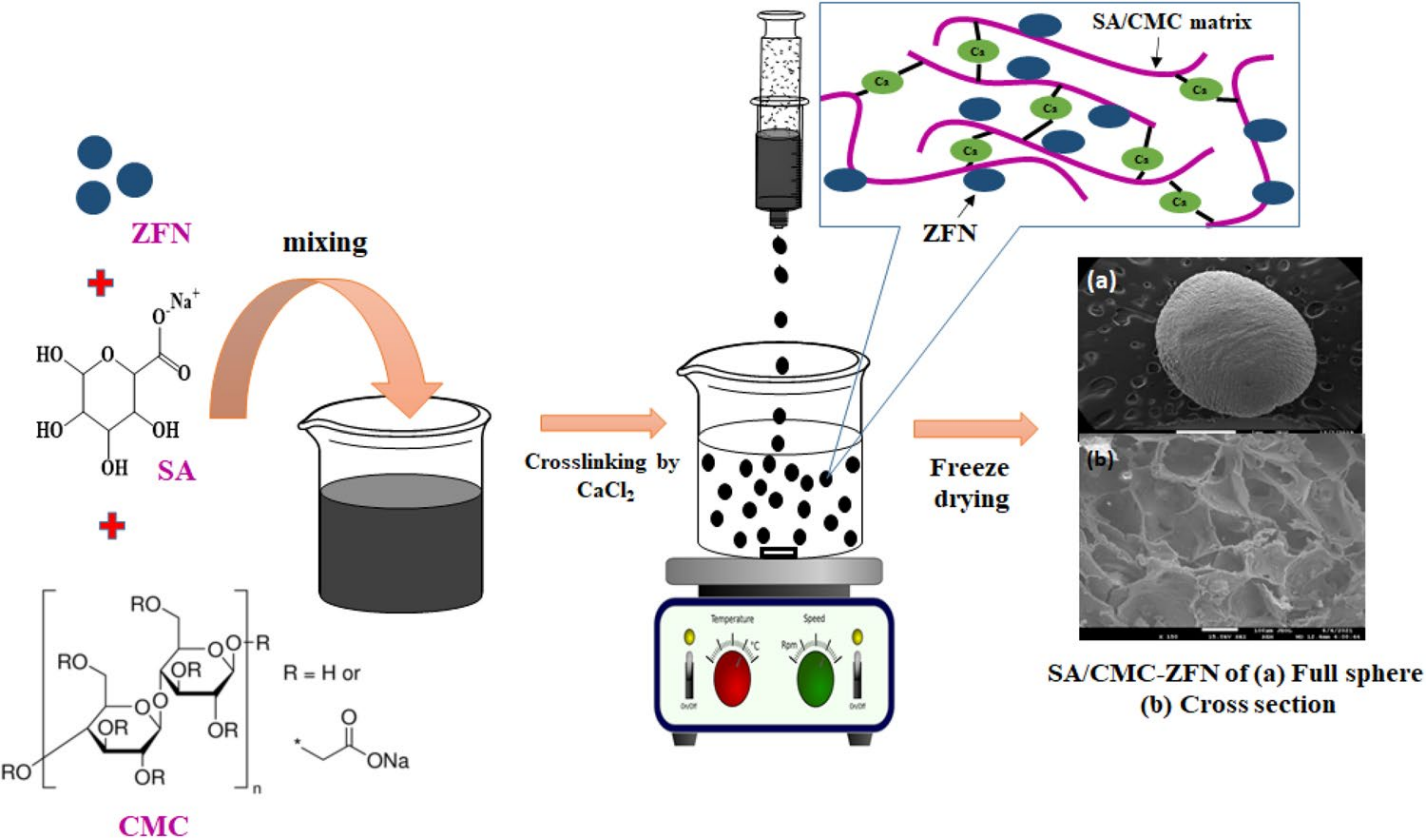
Diagrammatic representation of synthesis procedure of SA/CMC-ZFN.

Fly ash derived zeolite-stabilized nano zero-valent iron and nickel (ZFN) bimetallic composite was synthesized as described in our previous work^20^. Sodium alginate (SA)/Carboxymethylcellulose (CMC) entrapped ZFN (SA/CMC-ZFN) was fabricated via crosslinking with CaCl_2_, which was subsequently freeze-dried, as schematically illustrated in Fig. 1. Initially, SA (1 g) and CMC (1 g) were mixed in 100 mL water using a high-shear laboratory mixer until they completely dissolved. After that, 2 g of ZFN was added during polymer dissolution, with polymer and ZFN weight ratio at 1:1, and mixed to form a homogeneous solution. The slurry was subsequently added dropwise into a 0.5 M CaCl_2_ solution. After one hour, the resultant beads were detached from the CaCl_2_ solution and washed thoroughly with ethanol–water to eliminate excess CaCl_2_. They were finally freeze dried at − 80 °C for 48 h to obtain portable porous, feasible water treatment beads. Similarly, SA/CMC was synthesized but without the addition of ZFN. The chemicals and instruments used in this study for the characterization and their details are presented in the supplementary information.

### Adsorption conditions

Batch adsorption experiments were carried out to assess the performance of the produced SA/CMC-ZFN in terms of Cr(VI) and Cu(II) remediation. Initially, synthetic solutions contaminated Cu(II) and Cr(VI) were made by diluting 1000 mg/L stock solution of K_2_CrO_4_ and Cu(NO_3_)_2_.3H_2_O, respectively. During the experiment, the pH of the synthetic aqueous solution was controlled with 0.1 M or 1 M HNO_3_ or NaOH, and the ionic strength was regulated with 0.01 M NaCl. Typical batch adsorption studies were conducted in a 50 mL polyethylene falcon tube containing 50 mL of the required concentration of Cu(II) and Cr(VI) with 0.5 g/L of SA/CMC-ZFN at pH of 3 and 5, respectively. The residual content of various heavy metals in the filtrate was determined using an autosampler-equipped inductive coupled plasma – optical emission spectroscopy (ICP-OES, Avio 200, PerkinElmer, USA). The variable factors that impact heavy metal adsorptive removal such as initial metal concentration (10–200 mg/L for Cu(II) and 5–50 mg/L for Cr(VI)) and initial pH (2–8), contact time (15–1440 min) were investigated. Also, the effect of co-existing cations (Cd(II), Pb(II), Hg(II), and Co(II)), and anions (Cl^**−**^, SO_4_^2−^, NO_3_^−^, and F^**−**^) at concentrations of 50 and 20 mg/L for Cu(II) and Cr(VI) respectively, and temperature effect (25–45 °C), on the adsorption process of SA/CMC-ZFN were investigated systematically.

The following procedure was used to regenerate SA/CMC-ZFN. To begin, 0.5 g/L SA/CMC-ZFN was added to the heavy metal sample and agitated at 25 °C for 18 h before being removed from the solution. After that, the desorption tests were carried out by adding 0.1 M HNO_3_ to the adsorbent, followed by a rinsing with deionized water. Finally, the liquid-phase reduction approach by sodium borohydride was used to achieve the zero-valent form for Fe/Ni bimetallic particles of SA/CMC-ZFN. Following that, the regenerated SA/CMC-ZFN was used as an adsorbent.

### Real industrial wastewater- continuous column flow studies

To determine the performance of synthesized SA/CMC-ZFN for heavy metal removal on real time industrial water, samples were procured from the “Sihwa Banwol industrial complex, the Republic of Korea”. There samples of real-time industrial wastewater contained high concentration of heavy metal ions. The pH of the industrial wastewater was significantly lowered. The raw wastewater was adequately agitated before the adsorption experiment to achieve a uniform concentration. To improve adsorption effectiveness, the suspended matter was filtered through Whatman filter paper (42), and the pH of the filtered solution was adjusted to 3. Table 1 shows the composition and physical–chemical parameters of industrial effluent.

**Table 1:** Physico-chemical composition of industrial wastewater.

Parameters	Quantity in industrial wastewater	Parameters	Quantity in industrial wastewater
pH	2.05	Magnesium (mg/L)	3.66
Chromium (mg/L)	2.53	Sodium (mg/L)	39.81
Copper (mg/L)	3.95	Potassium (mg/L)	61.84
Nickel (mg/L)	51.96	Fluoride (mg/L)	150
Zinc (mg/L)	38.53	Chloride (mg/L)	740
Lead(mg/L)	0.43	Sulphates (mg/L)	650
Iron (mg/L)	4.1	Phosphate (mg/L)	766
Calcium (mg/L)	80.22		

Continuous column studies were performed in a glass cylinder with a diameter of 2 cm and a height of 11.5 cm, respectively. The adsorbent was packed into the glass column to achieve the desired bed height of 9.5 cm. To ensure tight packing of the adsorbent and prevent its loss, a layer of glass wool and sand was placed at the bottom and top of the column. This arrangement represent a packed bed column. All the experiments on the column are carried out at room temperature (25 °C). The peristaltic pump was then utilized to pump industrial effluent with a pH of 3 at a set flow rate of 0.2 mL/min. At regular intervals, the effluent was collected, and the residual concentration of heavy metals in industrial wastewater was determined.

## Results and discussion

### Material characterization studies

#### X-ray diffraction (XRD)

**Figure 2:**
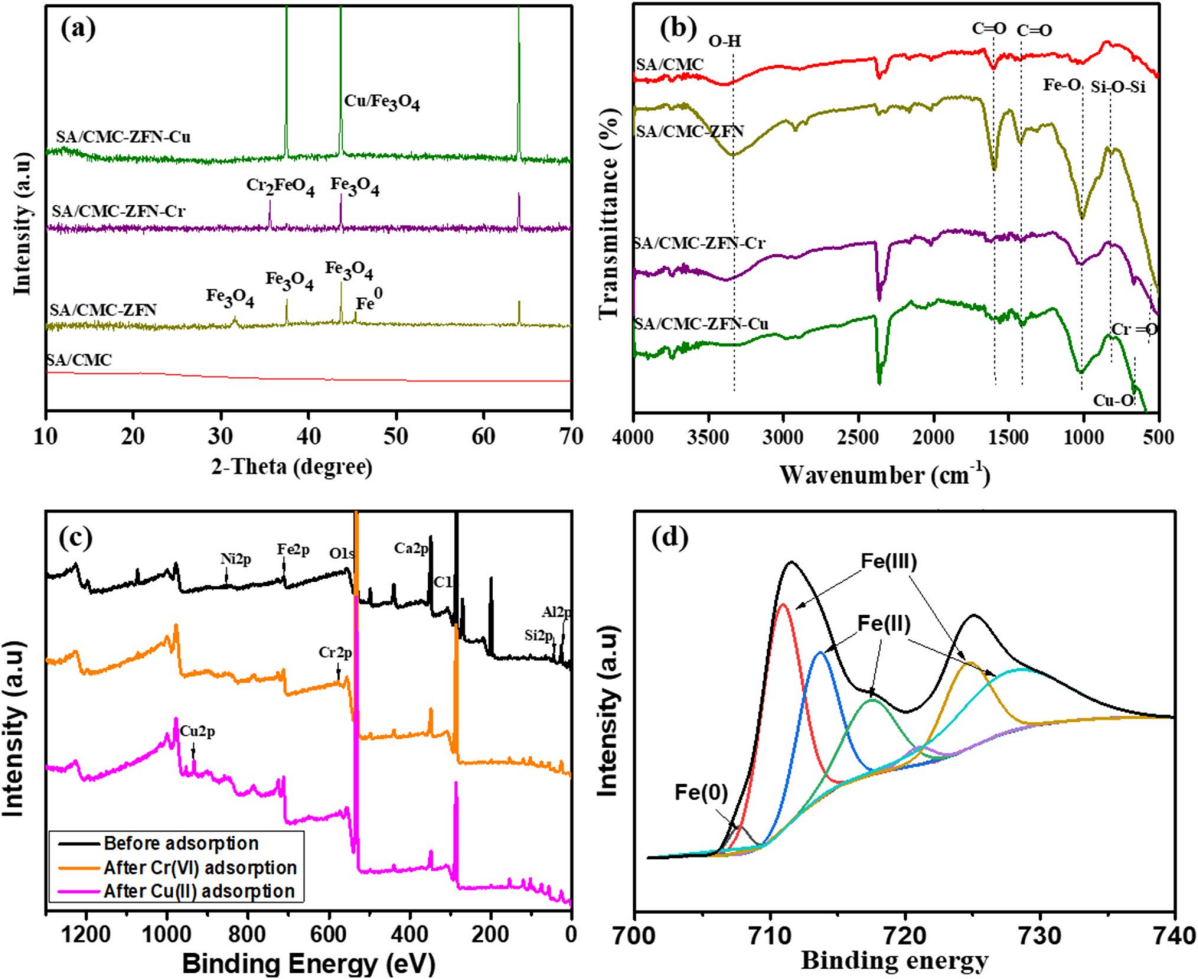
(**a**) X-ray diffraction pattern, and (**b**) Fourier transform infrared spectra of SA/CMC and SA/CMC-ZFN before and after adsorption, (**c**) XPS full scan of SA/CMC-ZFN before and after adsorption, (**d**) high-resolution XPS of Fe ion before adsorption.

The XRD patterns of SA/CMC and SA/CMC-ZFN before and after adsorption of Cr(VI) and Cu(II) is shown in Fig. 2a (the characterization findings of ZFN were given in Fig. S1). From these pattern, the crystallinity of Fe^0^ was confirmed at 2θ: 44.5°. The peak at 31.7° corresponds to Fe_2_O_3_ while the peaks 37.5°, 43.7° and 64.0° are related to Fe_3_O_4_^26^, confirming oxidation of iron and formation of iron oxide. The broad peak related to SA/CMC was around 21°^24^. It indicates that the ZFN nanoparticles successfully entrapped by SA/CMC polymer matrix. Due to the SA/CMC polymer matrix, zeolite-related peaks were not observed. After adsorption of Cr(VI) and Cu(II) on SA/CMC-ZFN was analyzed by XRD patterns (Fig. 2a) to understand the SA/CMC-ZFN texture changes. A detailed discussion of the adsorption mechanisms and texture changes upon metal adsorption/desorption are discussed in Removal mechanism and Regeneration studies section. The overall XRD results reveal the successful synthesis of SA/CMC-ZFN and application to remove Cu(II) and Cr(VI) from wastewater. The Brunauer-Emmet-Teller (BET) isotherm was used to determine the surface area of SA/CMC-ZFN, which is found to be 2.09 m^2^/g.

#### Fourier transform infrared spectra (FTIR) analysis

FTIR spectra of SA/CMC and SA/CMC-ZFN before and after adsorption were obtained in the range of 500–4000 cm^−1^ is shown in Fig. 2b. Several oxygen functional groups, such as in the range 3300–3400 cm^−1^ assigned to –OH (hydroxyl group) stretching vibrations and functional group –OH formed a broadband region at the wavenumber of 3300–3400 cm^−1^, the band range of 2700–2900 cm^−1^ indicating C = H. The peaks corresponding to –C = O was analyzed around 1600 cm^−1^ which confirms to presence of oxygen functional groups on the SA/CMC-ZFN surface, including carboxyl and epoxy. These are present in the polymer matrix of SA/CMC-ZFN^27^. Moreover, the Fe–O bonding vibration peak and symmetric vibration of Si–O–Si are at 1040 and 801 cm^−1^, respectively^28,29^. A detailed discussion of the mechanisms after adsorption are presented in Removal mechanism and Regeneration sections.

#### XPS analysis

XPS analysis was performed to explain the adsorption mechanism of heavy metals removal by SA/CMC-ZFN. Moreover, the chemical composition before and after treatment was assessed. The full XPS scan of SA/CMC-ZFN (Fig. 2c) displayed the peaks corresponding to Al, Si, C, O, Fe, and Ni, confirming the zeolite and Fe/Ni bimetallic composite^30^.

The peaks of Fe 2p_3/2_ and Fe 2p_1/2_ in the high-resolution spectra of Fe 2p is shown in Fig. 2d. These peaks explain the change of oxidization states of iron. A detailed discussion of the adsorption mechanisms is discussed in Sections “Removal mechanism” and “Regeneration”.

#### SEM–EDX analysis

**Figure 3:**
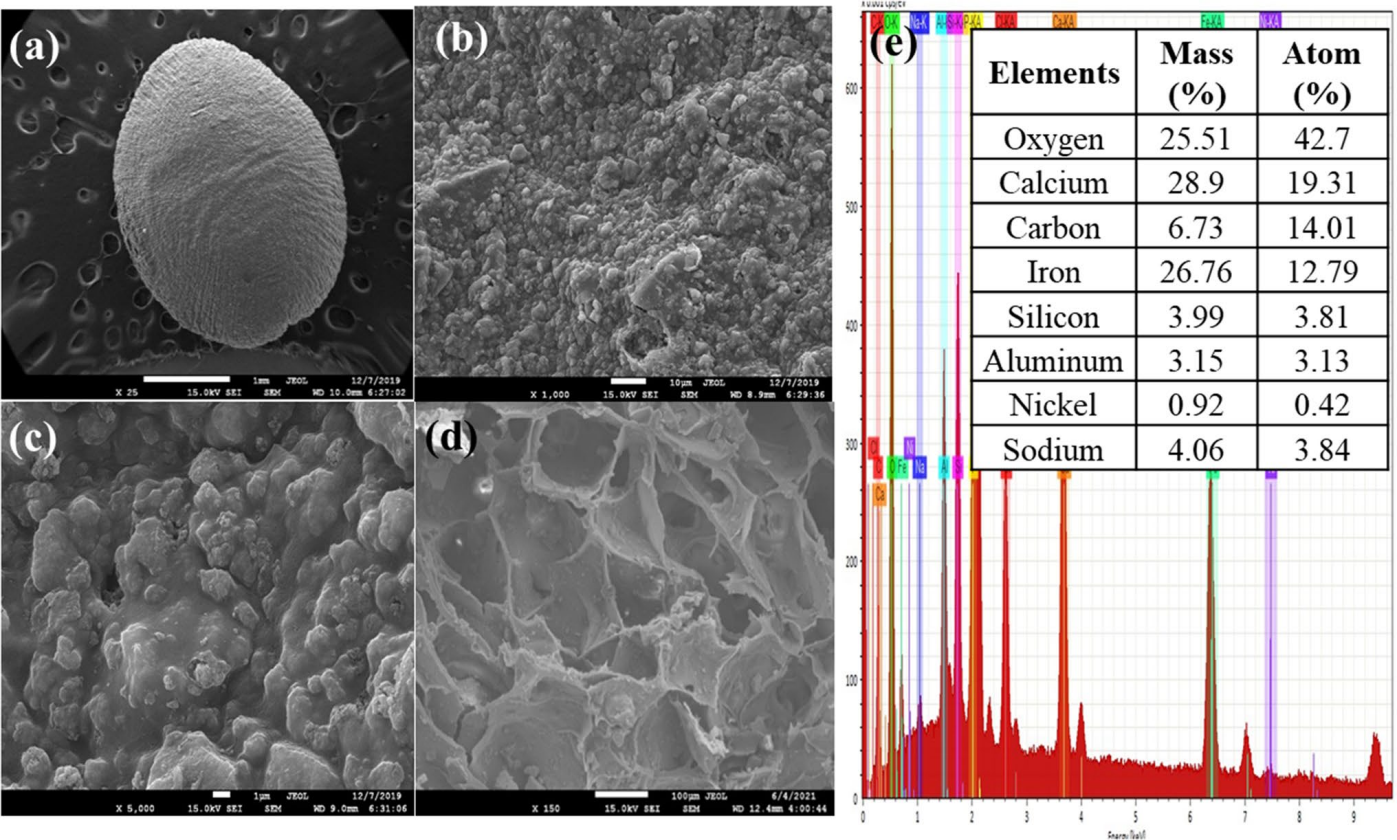
Scanning electron microscopy images (**a**) ×25, (**b**) ×1000, (**c**) ×5000, (**d**) cross section, of SA/CMC-ZFN; (**e**) EDX mapping of of SA/CMC-ZFN.

The surface morphology of porous SA/CMC-ZFN before and after adsorption is given in Fig. 3. The rough exterior surface of the SA/CMC-ZFN was encased in a uniform distribution of ZFN particles (Fig. 3a–c). It is proposed that the trapping of nZVI/Ni in SA/CMC can prevent particle aggregation. The cross-section shows the interior with high porosity and three-dimensional net structure of SA/CMC-ZFN (Fig. 3d). Figure 3e presents the EDX results of SA/CMC-ZFN, which observed the presence of main elements as oxygen, iron, and calcium were identified.

### Process parameters influence on adsorption experiments

#### pH effect

**Figure 4:**
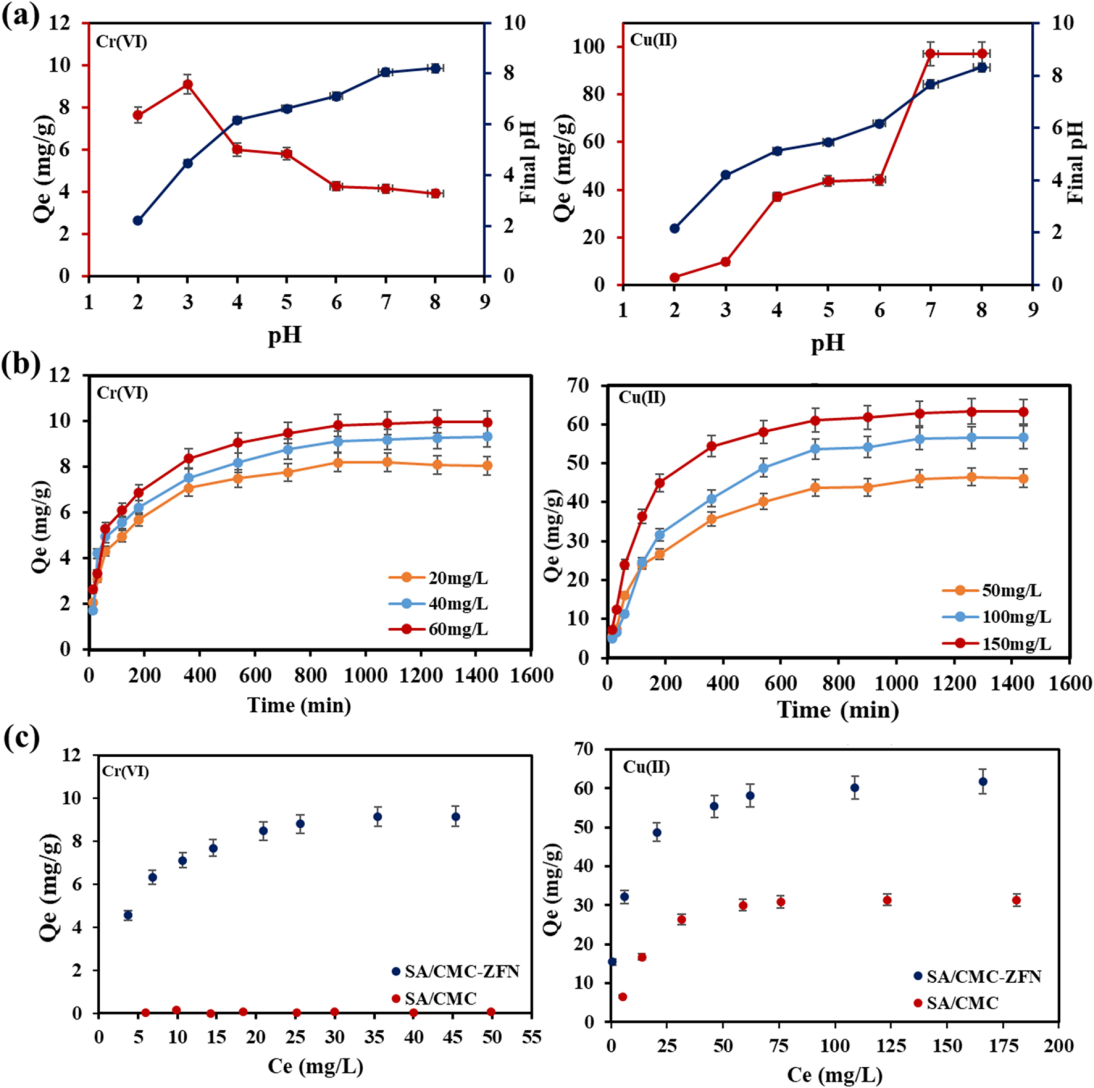
(**a**) Effect of pH: Experiment using SA/CMC-ZFN (Cu(II) = 50 mg/L, Cr(VI) = 20 mg/L), dosage = 0.5 g/L, Temp = 25 °C, IS = 0.01 M NaCl, pH = ∼2–8, RT = 18 h); (**b**) Effect of IC: Experiment using SA/CMC-ZFN, and SA/CMC (Cr(VI) (pH = 3, IC = 5–50 mg/L); Cu(II) (pH = 5, IC = 10–200 mg/L), IS = 0.01 M NaCl, RT = 18 h, dosage = 0.5 g/L, Temp = 25 °C); (**c**) Effect of Contact time: SA/CMC-ZFN (pH = 3, Cr (VI) IC = 20 mg/L); Cu(II) (pH = 5, IC = 50 mg/L), IS = 0.01 M NaCl, dosage = 0.5 g/L, Temp = 25 °C, RT = 15–1440 min); IC: Initial Concentration; RT: Reaction time, Temp: Temperature; IS: Ionic Strength.

The effect of solution pH on Cr(VI) and Cu(II) remediation was studied at a wide range of pH (2–8) is shown in Fig. 4a. The changes in adsorption % of Cr(VI) and Cu(II) at fixed pH were given in Fig. S2(a, b). Cr(VI) removal rapidly rose from pH of 2–3, then steadily increased. It might be driven by the fact that the functional groups on the adsorbent were easily protonated and were positively charged at low pH, making the SA/CMC-ZFN ideal for the adsorption of negatively charged HCrO4^−^. Furthermore, the reduction of Cr(VI) to Cr(III) by Fe^0^ was highly reliant on the pH of the solution because hydronium ions encourage Fe^0^ corrosion which Ni catalyzed and improved electron transport from SA/CMC-ZFN to Cr(VI), thus leading to surge in Cr(VI)^31,32^. Furthermore, the pHzpc of SA/CMC-ZFN is 8.75 (Fig.S2(c)). The final pH of Cr(VI) at initial pH of 3 would be 4.2; where SA/CMC-ZFN surface is a more positive charge which is seen from Fig.S2. This causes high adsorption of negative species of HCrO4^−^ of Cr(VI) at lower pH. At higher pH (> 6.0), Cr(VI) exist as CrO_4_^2−^ leads to repulsion by resulting negative surface obtained by enhanced amount of OH^−^ on the surface of SA/CMC-ZFN through the formation of iron hydroxide layer on the surface of the adsorbent, which prevented the electron transport from Fe^0^ to Cr(VI). These results are consistent with previously published works^32^.

However, at lower pH levels, the competition for reactive sites between H^+^ and Cu(II) would decrease Cu(II) adsorption^33^. The adsorption capacity rose gradually as solution pH increased, and most copper was removed under alkaline conditions. At higher pH, the copper ion is mostly found in moderately alkaline cationic complexes such as Cu(OH)^+^_,_ Cu_2_(OH)_2_^2+^, and Cu_2_(OH)_4_^2+^^34^. Considering the removal efficiency and final solution pH, optimum pH values for Cu(II) and Cr(VI) removal would be 3 and 5, respectively.

#### The influence of heavy metal concentrations and related isotherms

To understand the adsorption mechanism and capacity of the prepared nanocomposite adsorbent, the adsorption capacity of adsorbent (Qe) vs the metal equilibrium concentration (C_e_) was plotted and shown in Fig. 4b, and the data were represented in terms of Langmuir, Freundlich, and Temkin isotherm models. The removal of Cu(II) and Cr(VI) by SA/CMC-ZFN increased with concentration, perhaps due to the increased interference between the metal ions and reactive sites on the SA/CMC-ZFN surface. However, removal effectiveness was reduced at the high C_e_ of both metals due to a rapidly generated passive layer that slowed electron flow^32^.

The resultant correlation coefficient (R^2^) values (shown in Table 2 and Fig. S3) indicate that the adsorption of both metals on SA/CMC-ZFN is better explained by Langmuir isotherm than other models. The Q_max_ of SA/CMC-ZFN was 63.29 mg/g and 10.15 mg/g for Cu(II) and Cr(VI), respectively, higher than SA/CMC. Pristine SA/CMC did not exhibit affinity towards Cr(VI), which was consistent with the results of previous studies^35,36^. However, pristine SA/CMC has high adsorption capacity (34.48 mg/g) towards Cu(II). The overall results suggested that the SA/CMC-ZFN effectively removed Cu(II) and Cr(VI) from the aqueous solution. To comprehend the results obtained in this study, the Q_max_ of SA/CMC-ZFN for Cu(II) and Cr(VI) was compared with other adsorbents (Table 3). These results indicate that the Q_max_ of SA/CMC-ZFN was comparable, thereby signifying the potential use of this adsorbent in treating Cu(II) and Cr(VI) contaminated water.

**Table 2:** Adsorption isotherm model parameters for removing Cu(II) and Cr(VI) using SA/CMC-ZFN.

Metal ion	Adsorbent	Langmuir			Freundlich			Temkin		
		$\frac{{{\text{C}}_{{\text{e}}} }}{{{\text{Q}}_{{\text{e}}} }}{ } = { }\frac{1}{{\text{b}}}{ }\left( {\frac{1}{{{\text{K}}_{{\text{L}}} }}} \right){ } + { }\left( {\frac{1}{{\text{b}}}} \right){\text{C}}_{{\text{e}}}$			${\text{LogQ}}_{{\text{e}}} = {\text{LogK}}_{{\text{F}}} + \left( {\frac{1}{{\text{n}}}} \right){\text{LogC}}_{{\text{e}}}$			${\text{Q}}_{{\text{e}}} = {\text{B ln}}\left( {{\text{K}}_{{\text{T}}} } \right) + {\text{B ln}}\left( {{\text{C}}_{{\text{e}}} } \right)$		
		b (mg/g)	K_L_ (L/mg)	R^2^	K_F_ (mg/g)(L/mg)^1/n^	1/n (L/g)	R^2^	KT (L/mg)	B	R^2^
Cr(VI)	SA/CMC-ZFN	10.15	0.23	0.9994	3.55	3.68	0.9241	3.9	1328.1	0.9647
Cu(II)	SA/CMC-ZFN	63.29	0.2	0.9993	19.57	3.95	0.9571	9.62	282.8	0.9805
	SA/CMC	34.48	0.07	0.9942	4.68	2.4	0.831	0.74	350.5	0.9086

**Table 3:** Adsorption potential of various adsorbents for Cu(II) and Cr(VI) removal.

Adsorbent	Q_max_, mg/g		Conditions (pH, temperature and dose)	Refs
	Cu(II)	Cr(VI)		
nZVI on macroporous silica Foams	–	12.66	2, 25 °C, 1 g/L	^17^
Sodium Alginate-Polyethylene glycol oxide composite gel	5.6	–	4, 25 °C, 4 g/L	^8^
nZVI@SA/CMC-CA beads	52.51	–	4, 25 °C, 1 g/L	^10^
Chitosan modifies sodium dodecyl sulfate	–	3.23	4, 25 °C , 0.8 g/L	^37^
Fe_3_O_4_@Alginate beads	–	9.162	4.82, 30 °C, 2 g/L	^38^
Grape stalks entrapped into alginate beads	–	3.6	3, 20 °C , 42 g/L	^39^
Modified bagasse-cellulose	35.2	–	5, 30 °C, 0.5 g/L	^33^
Fe_3_O_4−_ alginate modified biochar	40.42	–	5, 25 °C, 2 g/L	^40^
SA/CMC-ZFN	–	10.15	3, 25 °C, 0.5 g/L	This study
SA/CMC-ZFN	63.29	–	5, 25 °C, 0.5 g/L	This study

#### Impact of contact time and adsorption kinetics

As shown in Fig. 4c, the removal capacity of Cu(II) and Cr(VI) increased with increasing contact duration and initial concentration. The removal rates of Cu(II) and Cr(VI) were rapid in the initial stages due to more empty active sites and higher Fe^0^ content along with high initial pollutants concentration. This illustrates the reduced capacity between Fe^0^, Cr(VI), and Cu(II) and the presence of strong affinity, which achieved equilibrium after 15 and 18 h, respectively. Considering this, for further studies, the optimal contact time of 18 h is considered for both heavy metals to further studies. The removal ability of Cu(II) and Cr(VI) by SA/CMC-ZFN increased with the concentrations of Cr(VI) and Cu(II). This is due to the increased likelihood of Cu(II) and Cr(VI) colliding with active sites on the SA/CMC-ZFN surface. Furthermore, the high concentrations of Cr(VI) and Cu(II) supplied the necessary driving force for resistances between the liquid and solid phases. In addition, SA/CMC showed a removal capability of around 27 mg/g for Cu(II) but low or no affinity for Cr (VI).

The pseudo 1st and 2nd order models were employed to evaluate the kinetics, as shown in Fig. S4, and the resulting data is provided in Table 4. Based on the correlation coefficient (R^2^) value of Cr(VI) and Cu(II), the pseudo second oder (PSO) model was notably the best at describing the kinetic data than the pseudo first oder (PFO) model and was closer to the experimental data. These adsorption kinetics results demonstrated that SA/CMC-ZFN adsorbed Cu(II) and Cr(VI) via chemisorption, which is the step controlling the efficiency of the adsorption process.

**Table 4:** Kinetic model parameters for removing Cr(VI) and Cu(II) using SA/CMC-ZFN.

Metal ion	Metal ion concentration (mg/L)	${\text{Q}}_{{\text{e}}}$, exp (mg/g)	Pseudo first order (PFO) ${\text{Log(Q}}_{{\text{e}}} - {\text{Q}}_{{\text{t}}} {)} = {\text{Log}}\,{\text{qQ}}_{{\text{e}}} - \left( {\frac{{{\text{k}}_{1} }}{2.03}} \right){\text{t}}$			Pseudo second order (PSO) $\frac{{\text{t}}}{{{\text{Q}}_{{\text{t}}} }}{ } = { }\frac{1}{{{\text{k}}_{2} {\text{Q}}_{{\text{e}}}^{2} }}{ } + \left( {\frac{1}{{{\text{Q}}_{{\text{e}}} }}} \right){\text{t}}$		
			${\text{Q}}_{{\text{e}}}$(mg/g)	k_1_ (min^−1^)	R^2^	${\text{Q}}_{{\text{e}}}$(mg/g)	k_2_(g/mg min)	R^2^
Cr(VI)	20	8.2	5.27	0.003	0.9806	8.54	0.001	0.9988
	40	9.11	6.02	0.003	0.9783	9.81	0.001	0.9984
	60	9.9	6.63	1.89	0.9917	10.5	0.001	0.9991
Cu(II)	50	46.04	53.5	0.003	0.9881	51.54	1.32E−04	0.9986
	100	56.32	37.7	0.003	0.9796	67.11	6.57E−05	0.9949
	150	62.83	49.72	0.004	0.9906	68.96	1.31E−04	0.9992

#### Effect of co-existing ions

**Figure 5:**
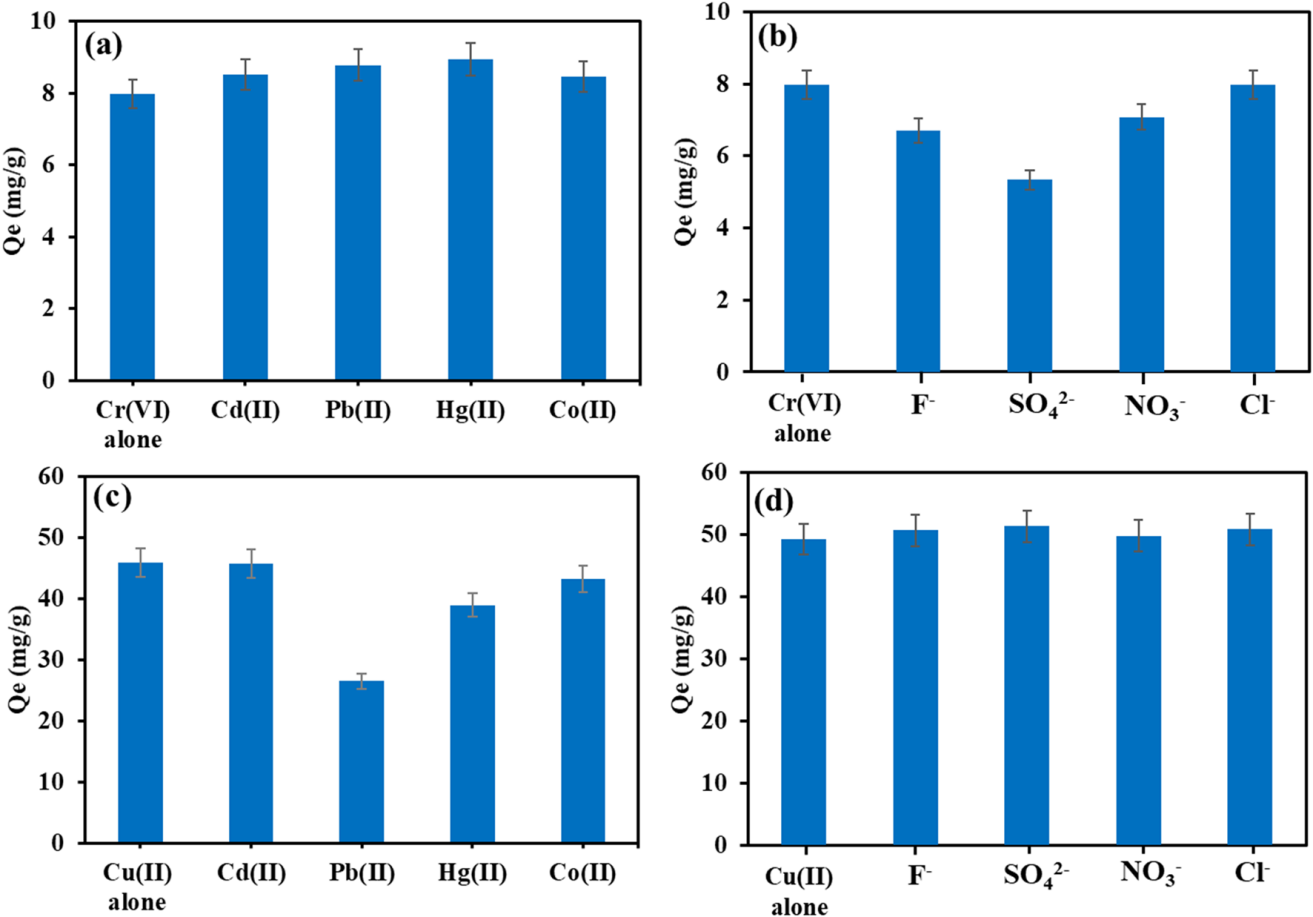
(**a**, **b**) Effect of co-existing cations, (**c**, **d**) effect of co-existing anions on Cu(II) and Cr(VI) removal using SA/CMC-ZFN, ((pH = 3, Cr(VI) = 20 mg/L) (pH = 5, Cu(II) = 50 mg/L), dosage = 0.5 g/L, reaction time = 18 h, temperature = 25 °C).

From Table 1, it was found that aside from Cr(VI) and Cu(II), the effluent was also high in nickel and zinc. Other cations and anions present in large concentrations in industrial effluent include sodium, pottasium, calcium, magnesium, F^─^, Cl^─^, SO_4_^2─^, and PO_4_^3─^. Hence, the effect of anions (F^─^, SO_4_^2−^, NO_3_^−^, and Cl^**−**^**)** and cations (Cd^2+^, Pb^2+^, Hg^2+^, and Co^2+^) on the removal of both metals were examined (as shown in Fig. 5). The results indicate that the co-existing cations had minimal influence on Cr(VI) removal; while anions did not affect Cu(II) removal. However, in the presence of F^─^ and NO_3_^−^, Cr(VI) elimination was only moderately affected and considerably decreased in the presence of SO_4_^2−^. The competitive adsorption of Cr(VI) ions and SO_4_^2−^ at the surface active sites may lower Cr(VI) removal. The presence of Cl^**−**^ did not affect the removal of Cr(VI). The co-existing cations reduced the adsorption of Cu(II) slightly; but Pb(II) substantially reduced the removal of Cu(II) by SA/CMC-ZFN.

#### Thermodynamics

The thermodynamics studies were conducted to understand the energy variations and inherent mechanisms of adsorption process. The thermodynamics parameters such were calculated using the following equations.

1 $$\Delta {\text{G}}^\circ = - {\text{RT}}\;{\text{LogKc}}$$

2 $$\Delta {\text{G}}^\circ = \Delta {\text{H}}^\circ - {\text{T}}\Delta {\text{S}}^\circ$$

Based on the preceding equations, plots were drawn for both metal ions, as shown in Fig. S5. ΔH° and ΔS° can estimate from slope and intercepts. The values of ΔH°, ΔS°, and ΔG° calculated using the above equations are provided in Table 5. The negative ΔG° values indicate the spontaneity of the Cu(II) and Cr(VI) adsorption process using SA/CMC-ZFN. In addition, the ΔG° value decreases with the increasing temperature, which depicts the feasibility of higher temperatures in the adsorption of Cu(II) and Cr(VI) ions on the SA/CMC-ZFN. The presence of a positive value of H indicated that the adsorption reaction for both Cu(II) and Cr(VI) was endothermic, implying that high temperatures were preferred for improved removal of both ions by SA/CMC-ZFN. Moreover, the change in ∆H° was found to be positive, suggesting that more disorder was generated at the solid-solution interface than Cu(II) and Cr(VI) removal. The positive ΔS° corresponds to an increase in the degree of randomness at the liquid–solid interface^10,41^.

**Table 5:** Thermodynamic parameters of Cu(II) and Cr(VI) removal by SA/CMC-ZFN.

	Temperature (°C)	Q max	ln K_c_	∆G° (KJ/mol)	∆H° (KJ/mol)	∆S° (J/K/mol)
Cr(VI)	298	10.15	0.84	− 2.1	5.46	25.45
	318	14.01	1.01	− 2.68		
	338	15.34	1.1	− 3.11		
Cu(II)	298	63.29	2.54	− 6.3	2.09	28.17
	318	71.43	2.59	− 6.85		
	338	75.19	2.64	− 7.42		

#### Removal mechanism

**Figure 6:**
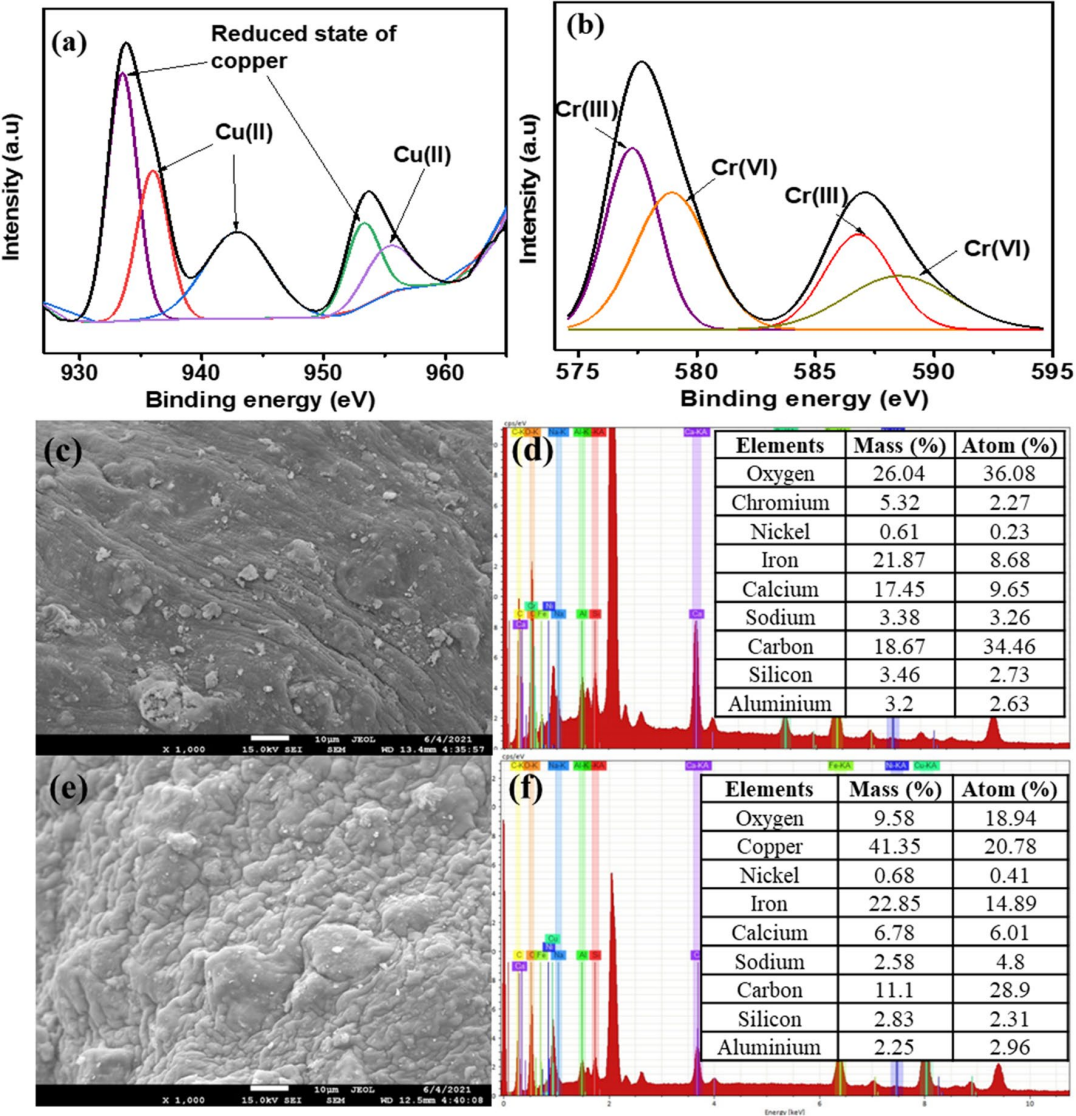
XPS analysis of SA/CMC-ZFN after adsorption of (**a**) Cu(II), and (**b**) Cr(VI); SEM–EDX elemental composition of SA/CMC-ZFN after adsorption of (**c**, **d**) Cr(VI), and (**e**, **f**) Cu(II).

The comprehensive XPS spectra for Cr 2p and Cu 2p were obtained to investigate the chemical compositions of Cu and Cr. In Fig. 6a, the peaks at 932.3 and 952.9 eV could be ascribed to Cu 2p_3/2_ and Cu 2p_1/2_, respectively, reflecting a reduced state of copper (Cu(0) or Cu(I))^10,42^. It meant that the Cu(II) had been reduced to Cu(0) or Cu (I). The other peaks at 935.6 and 955.4 eV characterize the energies of 2p_3/2_ and 2p_½_, respectively, which are attributed to Cu(II) in Cu(II) oxide/hydroxide^43,44^. These results suggest that the copper ions could be removed using SA/CMC-ZFN by surface precipitation and reduction (Cu(II) to Cu(0) or Cu(I)). Figure 6b depicts the high-resolution XPS for Cr 2p after adsorption. The Cr 2p XPS spectra revealed four main peaks, with the binding affinity of 577.2 and 586.8 eV correlating to Cr(III) 2p_3/2_ and 2p_1/2_, respectively, indicating Cr(OH)_3_, Cr(OH)O, and Cr_2_O_3_. However, the other peaks at 578.9 and 588.7 eV were assigned to Cr(VI) 2p_3/2_ and 2p_1/2_, respectively^41,45,46^, indicating that Cr(VI) and Cr(III) co-existed on the SA/CMC-ZFN following adsorption. The existence of a high-intensity signal of Cr(III) showed that Cr(VI) was reduced to Cr(III) throughout the adsorption process and that Cr(III) is the most predominant chromium species on the SA/CMC-ZFN. Furthermore, the elimination of Cr(VI) is related to the redox interaction between nZVI and Cr(VI), which produces Cr(III) and Fe(III). Because H^+^ is used in this reduction process, Cr(III) and Fe(III) can interact with excess OH^−^ to generate co-precipitation products such as Cr(OH)_3_, Fe(OH)_3_, or Cr_x_ Fe_1−x_(OH)_3_^46^. Overall, the results indicate that Cr(VI) was reduced to a less hazardous Cr(III) during adsorption, which stabilized and precipitated as oxy/hydroxide forms on the SA/CMC-ZFN.

The SEM images (Fig. 6(c, e)) revealed that the smooth surface morphology was observed on the surface of the SA/CMC-ZFN due to heavy metal adsorption. Chromium and copper were identified in the SA/CMC-ZFN EDX elemental composition (Fig. 6(d, f)). The quantity of calcium reduced following Cr(VI) and Cu(II) adsorption, implying cation exchange between Ca(II) ions and Cu(II)/Cr(III) ions. These results revealed that the ZFN nanocomposite was successfully entrapped in the SA/CMC polymer matrix, and Cu(II) and Cr(VI) were significantly removed from the contaminated solution.

**Figure 7:**
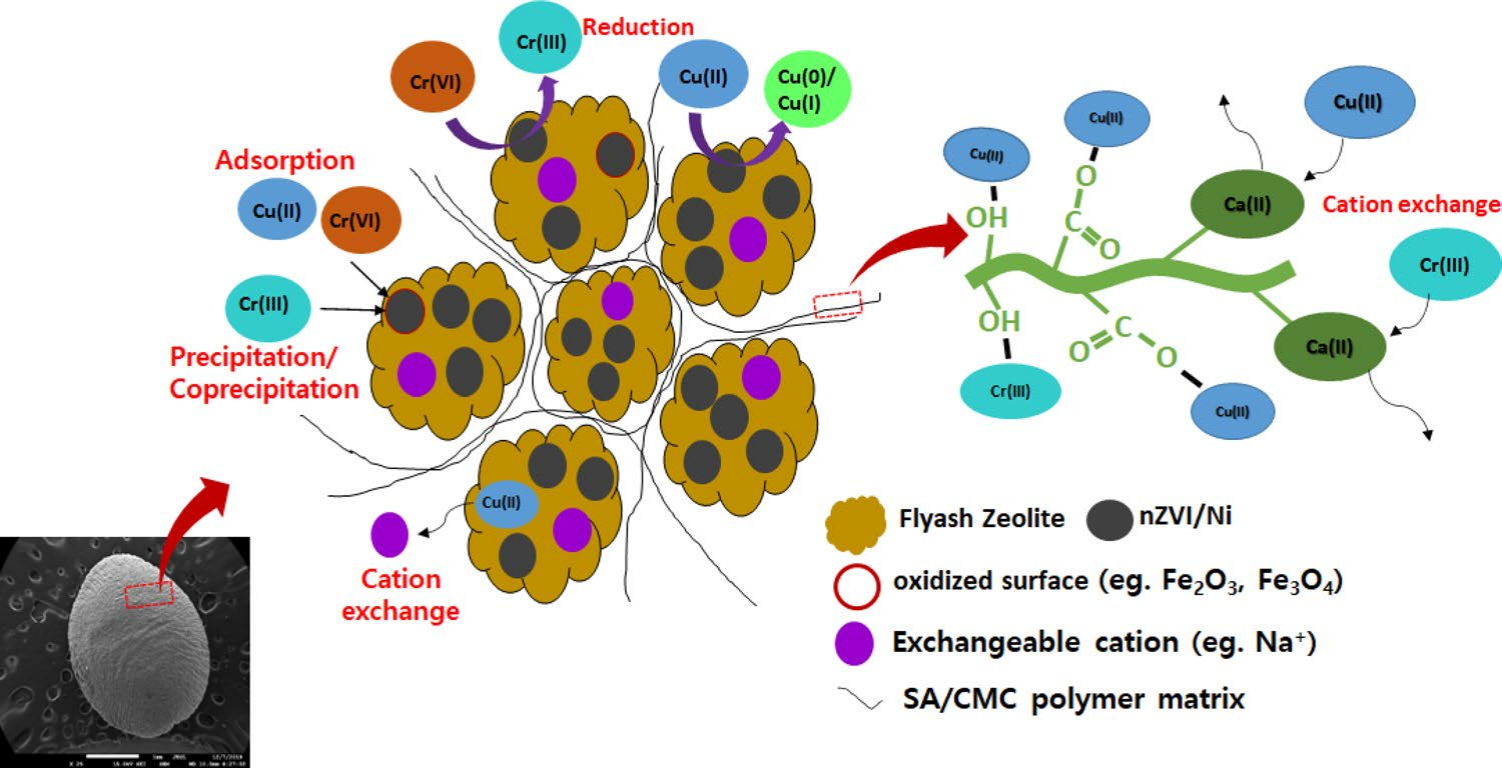
Diagrammatic representation of Cu(II) and Cr(VI) removal from solution by SA/CMC-ZFN.

The overall findings suggest that the adsorption mechanism, the Cu(II) and Cr(VI) removal using SA/CMC-ZFN are influenced by various mechanisms, including reduction, adsorption, precipitation, and ion exchange, according to XPS, SEM–EDX, FTIR, and XRD investigations (Fig. 7). The metal ion in the solution initially reached the proximity of the SA/CMC-ZFN and adsorbed; subsequently, the nZVI/Ni interacts with the heavy metal and is reduced by being oxidized in the solution. Fe^0^ provides electrons to Cr(VI), which is catalyzed by Ni, which reduces Cr(VI) to Cr(III), followed by adsorption, precipitation as hydroxides and/or (oxy) hydroxides^31,32^. Moreover, Cr(III) can be co-precipitated on the iron oxide, which is present on the surface of nZVI, by interacting with Fe(III) and OH^−^ to produce Fe(OH)_3_, Cr(OH)_3_, or Cr_x_ Fe_1−x_(OH)_3_^46^. Cu(II) is reduced to Cu(0)/Cu(I) by supplying electrons via nZVI; the iron oxide layer adsorbs it directly. Furthermore, Cr(III) and Cu(II) were eliminated by zeolite by its cation exchange capacity, SA/CMC matrix by exchanging with Ca(II), and by interacting with the -OH and COO^−^ functional groups which are on the SA/CMC-ZFN surface^10,39,47^.

#### Regeneration

**Figure 8:**
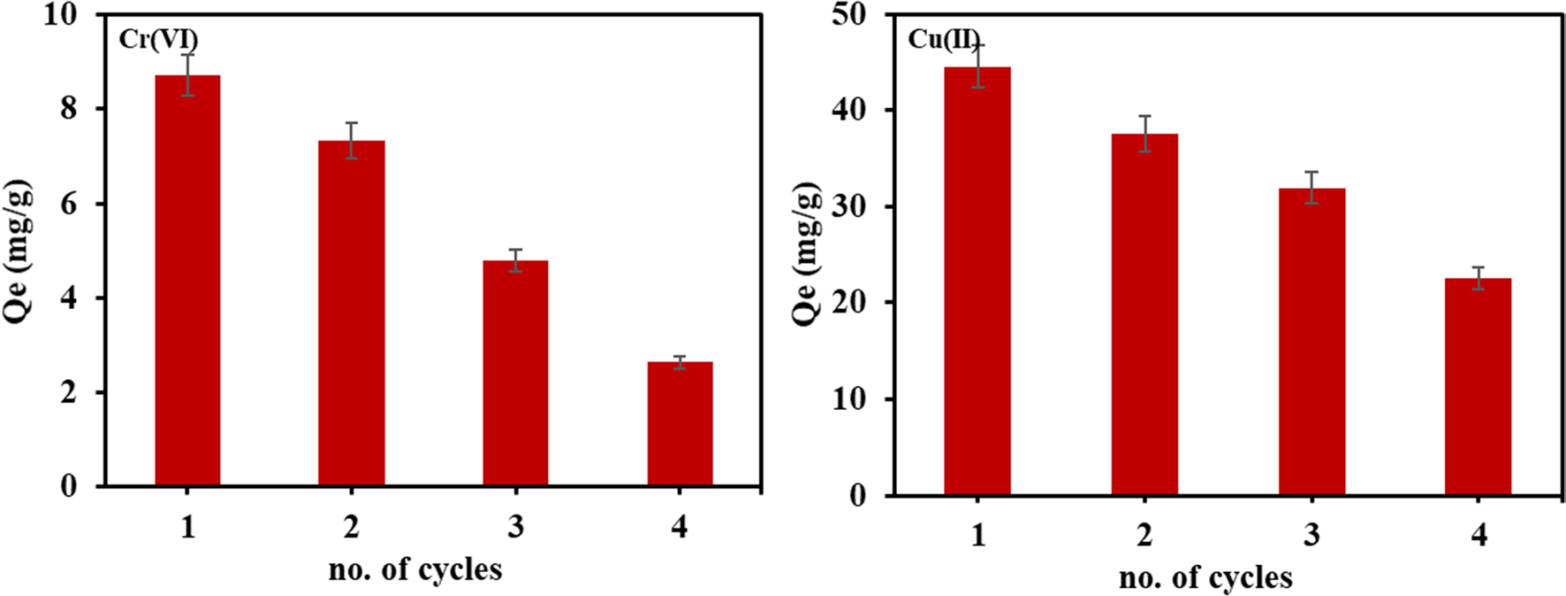
Regeneration of SA/CMC-ZFN for Cu(II) and Cr(VI) removal.

**Figure 9:**
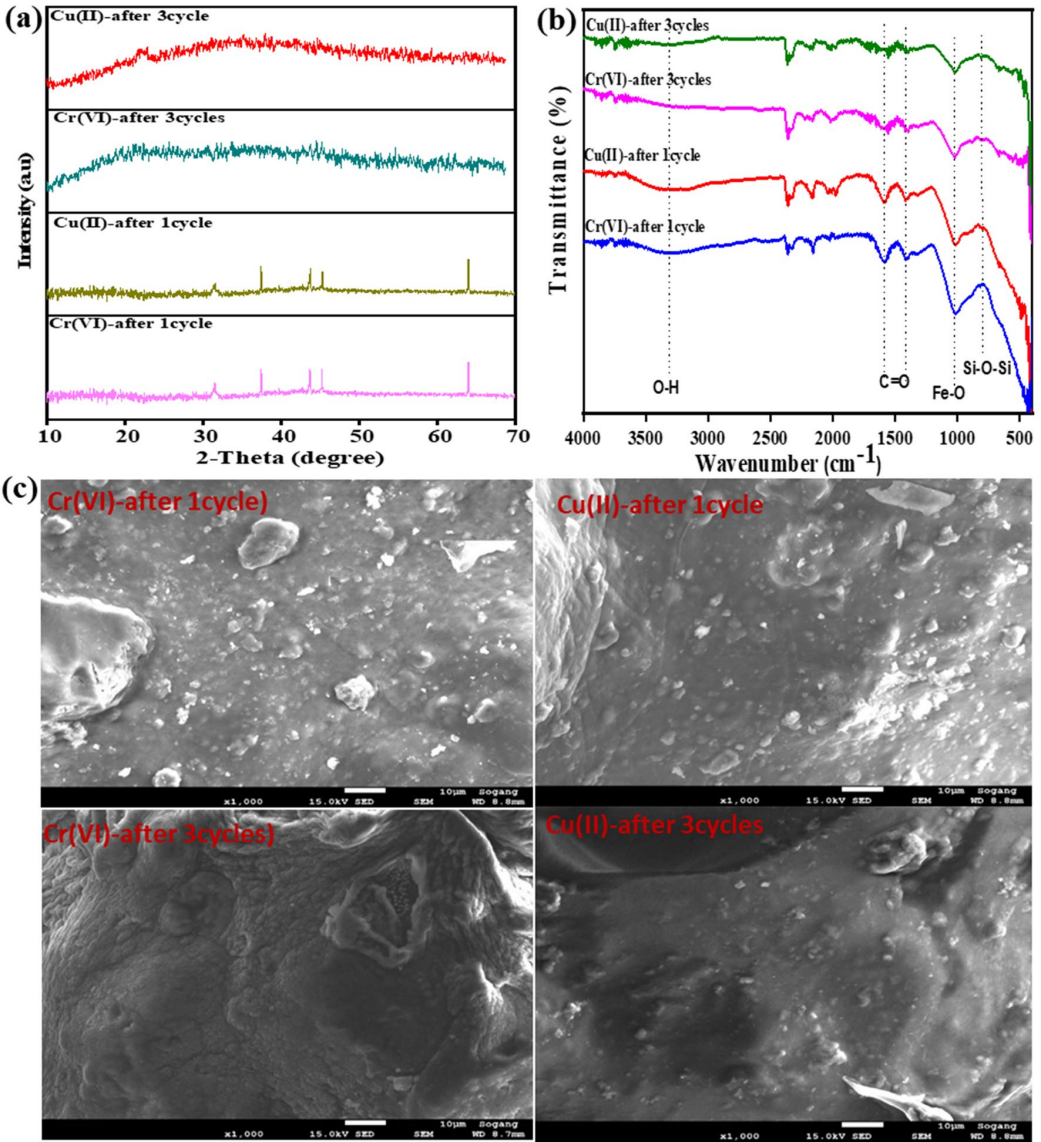
(**a**) X-ray diffraction pattern, and (**b**) Fourier transform infrared spectra of SA/CMC and SA/CMC-ZFN, (**c**) SEM images of regenerated SA/CMC-ZFN.

The regeneration is very important in assessing the cost-effectiveness of adsorbent as well as reducing the huge generation of adsorbents. Figure 8 depicts the regeneration of SA/CMC-ZFN after treating the Cu(II) and Cr(VI) contaminated water. The removal capability of SA/CMC-ZFN towards the heavy metals ions was steadily diminished as the number of cycles increased. Figure 9a, b shows the XRD and FT-IR of regenerated adsorbent; where the intensity of iron-related peaks and the functional groups which are on the surface of the adsorbent were gradually decreased with the increasing number of cycles. Hence, the surface morphology of SA/CMC-ZFN was affected (Fig. 9c). Further, Fig. 2a confirmed that the disappearance of Fe^0^ upon the adsorption of heavy metals is due to adsorption of Cr(VI) and Cu(II) on Fe^0^ and conversion to iron oxide^27–29^. Additionally, the change of surface functional groups was interpreted from Fig. 2b, where the fresh peaks at 569 cm^−1^ and 662 cm^−1^ were considered to be Cr = O and Cu–O, respectively^48,49^. These peaks appeared after adsorption of Cr(VI) and Cu(II) on the surface of SA/CMC-ZFN. The intensity of oxygen functional groups decreased after adding Cr(VI) and Cu(II). These findings indicate that the surface functional groups of SA/CMC-ZFN play an important role in removing Cr(VI) and Cu(II) from an aqueous solution. The oxidation of Fe^0^ upon adsorption of heavy metals on SA/CMC-ZFN was clearly understood from Fig. 2d, where strong oxygen peaks exist due to the oxidation of iron. New Cr(VI) and Cu(II) peaks emerged on the surface scan of SA/CMC-ZFN upon Cr(VI) and Cu(II) adsorption. The peaks Fe 2p_3/2_ and Fe 2p_1/2_ in the high-resolution spectra of Fe 2p (Fig. 2d) represented photoelectron peaks at 711.1 eV and 724.8 eV, respectively. These peaks indicates the Fe(III) species in the SA/CMC-ZFN contain a conceivable chemical structure of ferric hydrate (FeOOH), magnetite (Fe_3_O_4_), or hematite (Fe_2_O_3_). Furthermore, shakeup satellite peaks at 707.3 eV ascribed to Fe(II) were responsible for Fe^0^^46,50^. Furthermore, the substantial changes in the adsorbent’s elements content are depicted in Fig. S6. Moreover, a tiny amount of adsorbate remained on the active sites of the SA/CMC-ZFN surface pores.

To understand the changes in surface area or pore volume with adsorption–desorption cycles, BET is investigated. The BET surface area of SA/CMC-ZFN was found to be 2.09 m^2^/g. It was changed slightly in the regenerated composite after 1 cycle towards Cr(VI) and Cu(II). In contrast, it was increased remarkably after 3 cycles of regeneration to 6.61 and 8.38 m^2^/g for Cr(VI) and Cu(II), respectively (Table 6). These results indicated that the loss of iron and other exchangeable ions during the desorption process leads to increased pore volume along with increased surface area. Further, it leads to declining of adsorption capacity with regenerated cycles. Despite this, the SA/CMC-ZFN could reuse up to 4 cycles for Cr(VI) and Cu(II). The overall regenerated composite findings concluded the cost-effective application of SA/CMC-ZFN to remove Cu(II) and Cr(VI) from aquatic solutions.

**Table 6:** BET surface area and pore volume of SA/CMC-ZFN.

	BET surface area (m^2^/g)	Total pore volume (cm^3^/g)
Before adsorption	2.09	0.0051
Cr(VI) after 1 cycle	1.22	0.0046
Cu(II) after 1 cycle	2.94	0.0047
Cr(VI) after 3 cycles	6.61	0.0099
Cu(II) after 3 cycles	8.38	0.0103

## Comparison of the economic feasibility of SA/CMC-ZFN with commercial natural zeolite

According to the experimental results conducted on contaminated water, the adsorption performance of SA/CMC-ZFN and commercial natural zeolite was confirmed to be 10.47 and 0.46 mg/g, respectively. The unit cost of treating 1 ton of contaminated water and 1 kg for each adsorbent were calculated based on the adsorption performance. As shown in Table 7, the price of 1 kg of commercial natural zeolite and SA/CMC-ZFN are 5000 and 29,269 KRW, respectively, and the unit cost of treating 1 ton of contaminated water using them was 111,780 and 28,098 KRW, respectively. The reduced ability of SA/CMC-ZFN towards Cr(VI) decreases the cost of the adsorbent consumed for reduction and has a higher adsorption performance than commercial natural zeolite. Hence, SA/CMC-ZFN was predicted to have four times higher economic efficiency (approximately). In addition, the unit cost of the coagulation/sedimentation method (which is a conventional treatment method of the existing plating wastewater), is approximately 100,000 KRW per ton. Therefore, the treatment of plating wastewater using the SA/CMC-ZFN is expected to have an economic efficiency that is 3.6 times higher than the coagulation/sedimentation method.

**Table 7:** Cost comparison of SA/CMC-ZFN versus commercial zeolites.

Adsorbent	Commercial zeolite	SA/CMC-ZFN
1 kg price	5000 KRW	29,269 KRW
Heavy metal adsorption performance	0.46 mg/g	10.47 mg/g
Amount of adsorbent required to treat 1 ton of polluted wastewater (Plating wastewater with a sum of heavy metal concentrations of 10 mg/L)	Approximately 21.7 kg	Approximately 0.96 kg
adsorbent price	108,700 KRW	28,098 KRW
Reductant (sodium metabisulfite) price (100 mg/L input)	3080 KRW	–
1 ton processing unit price	111,780 KRW	28,098 KRW

## Outcomes of continuous column treatment of real industrial wastewater and a comparative study with commercial zeolite

The adsorbent performance was evaluated using the Thomas, Adams-Bohart, and Yoon-Nelson model, a set of model equations that can predict the results obtained from the continuous column experiment, the breakthrough curve of the column reactor, and evaluate the efficiency^51–53^. The Thomas model is the most widely used to characterize continuous adsorption systems and no axial dispersion. In addition, the Thomas constant and the maximum adsorption capacity can be calculated, which is used to predict the breakthrough curve. The Thomas model equation is described as follows:

3 $${\text{Ln}}\left( {\frac{{{\text{C}}_{{\text{o}}} }}{{{\text{C}}_{{\text{t}}} }}} \right)( - 1) = \frac{{{\text{k}}_{{{\text{Th}}}} {\text{q}}_{{\text{o}}} {\text{M}}}}{{\text{Q}}} - {\text{ k}}_{{{\text{Th}}}} {\text{C}}_{{\text{o}}} {\text{t}}$$

Here, k_Th_ (mL/min mg) is the Thomas constant, q_0_ (mg/g) is the maximum adsorption performance per unit mass, M(g) is the mass of the adsorbent, and Q(min/L) is the flow rate.

The Adams-Bohart model is mainly used to describe the initial part of the breakthrough curve. It assumes that the adsorbent capacity and its concentration govern the adsorption rate. The Adams-Bohart model equation is as follows.

4 $${\text{ln}}\left( {\frac{{{\text{C}}_{{\text{t}}} }}{{{\text{C}}_{{\text{o}}} }}} \right) = {\text{k}}_{{{\text{AB}}}} {\text{C}}_{{\text{o}}} {\text{t}} - {\text{ k}}_{{{\text{AB}}}} {\text{N}}_{0} \left( {\frac{{\text{Z}}}{{\text{F}}}} \right)$$

where k_AB_ (mL/min mg) is the Adams-Bohart constant, and N_0_ (mg/cm^3^) is the maximum adsorption performance per unit volume. Z(cm) is the height of the column, and F(cm/min) is the linear velocity of the discharged treated water.

The Yoon-Nelson model implies that the rate at which the adsorption potential of each adsorbate molecule declines is proportional to the adsorption potential and the breakthrough potential of the adsorbate. This model is simple and does not require detailed information about the column system. The expression of the Yoon-Nelson model is as follows.

5 $$\ln \left( {\frac{{{\text{C}}_{{\text{t}}} }}{{{\text{C}}_{{\text{o}}} - {\text{C}}_{{\text{t}}} { }}}} \right) = {\text{ k}}_{{{\text{YN}}}} -\uptau {\text{k}}_{{{\text{YN}}}}$$

where k_YN_ (min^−1^) is the rate constant, and τ(min) is the time it takes for the adsorbate to break through 50%.

The error (sum square) between the actual breakthrough curve and the breakthrough curve predicted by each model was compared, which was used to identify the best model for predicting the breakthrough curve among the models employed. The formula for the error of each model is as follows:

6 $${\text{SS}} = \frac{{\sum \left[ {\left( {\frac{{{\text{C}}_{{\text{t}}} }}{{{\text{C}}_{0} }}} \right)_{{\text{c}}} - \left( {\frac{{{\text{C}}_{{\text{t}}} }}{{{\text{C}}_{0} }}} \right)_{{\text{e}}} } \right]^{2} }}{{\text{N}}}$$

Here, (C_t_/C_0_)_c_ represents the value predicted by the model, and (C_t_/C_0_)_e_ represents the value obtained by the experiment.

**Figure 10:**
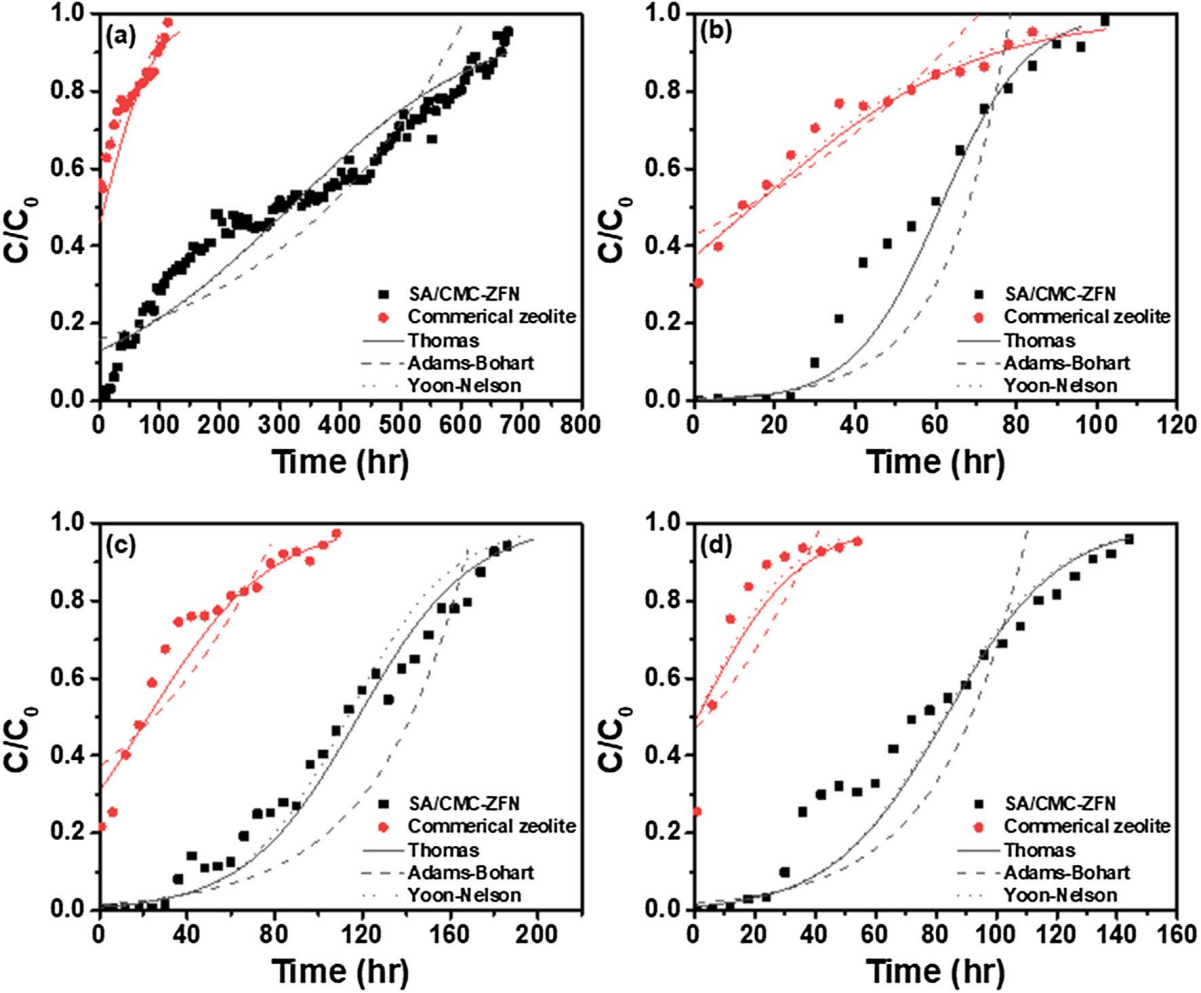
Breakthrough curves for adsorption of heavy metals onto SA/CMC-ZFN and commercial zeolite. (**a**) Chromium; (**b**) copper; (**c**) zinc; (**d**) nickel.

From Table 1, it was found that aside from chromium and copper, the effluent was also high in nickel and zinc; hence, the zinc and nickel removal studies were also conducted with the present material in the column studies. The experiment results for continuously treatment of contaminated wastewater using a column reactor loaded with SA/CMC-ZFN depicted in Fig. 10. Due to the high dose of SA/CMC-ZFN used to extract the adsorbate, the final pH of the adsorbate would be greater than 7. It may be due to alkaline precipitation. Hence, the continuous column experiment was conducted at pH of 3 for all heavy metal ions. Heavy metals in the contaminated wastewater rapidly ruptured the commercial natural zeolite shortly after the column reactor began operating. After the heavy metal removal, the commercial zeolite was confirmed to break gradually. However, the column reactor filled with SA/CMC-ZFN remained stable for approximately 36 h. In order to select the processing performance of the adsorbed material in the column reactor and an appropriate breakthrough prediction model, the results obtained in the continuous processing experiment were substituted into Thomas, Adams-Bohart, and Yoon-Nelson models. The parameters were calculated for each model (Table 8). Table 9 displays the sum of square error calculation outcomes for each model after comparing the difference between the predicted and actual breakthrough curves.

**Table 8:** Parameters of Thomas, Adams-Bohart, Yoon-Nelson model of continuous treatment result of real industrial wastewater.

Elements	Materials	C_0_ (mg/L)	pH	Velocity (cm/min)	Thomas		Adams-Bohart		Yoon-Nelson	
					k_Th_ (L/mg·min)	q_0_ (mg/g)	k_AB_ (L/mg·min)	N_0_ (mg/cm^3^)	k_YN_ (min^−1^)	τ (min)
Copper	SA/CMC-ZFN	3.94	3	0.05	4.05.E−04	0.26	2.78E−04	0.12	1.60.E−03	3667
	Commercial zeolite				1.52.E−04	0.03	5.06E−05	0.11	6.32.E−04	821
Nickel	SA/CMC-ZFN	51.96	3	0.05	1.92.E−05	4.72	1.15E−05	2.32	9.00.E−04	4942
	Commercial zeolite				1.92.E−05	0.03	5.77E−06	0.88	1.10.E−03	61
Zinc	SA/CMC-ZFN	47.79	3	0.05	1.82.E−05	4.96	1.04E−05	2.65	7.00.E−04	6795
	Commercial zeolite				1.56.E−05	0.39	5.19E−06	1.28	6.00.E−04	1337
Chromium	SA/CMC-ZFN	2.33	3	0.05	6.52.E−05	0.53	3.26E−05	0.38	1.00.E−04	19,113
	Commercial zeolite				2.61.E−04	0.01	4.56E−05	0.07	4.00.E−04	440

**Table 9:** Comparison of sum square error of each model.

Elements	Materials	Thomas	Adams-Bohart	Yoon-Nelson
Copper	SA/CMC-ZFN	0.007	0.185	0.007
	commercial zeolite	0.151	0.102	0.102
Nickel	SA/CMC-ZFN	0.005	0.113	0.005
	commercial zeolite	0.002	0.037	0.025
Zinc	SA/CMC-ZFN	0.015	0.049	0.002
	commercial zeolite	0.001	0.053	0.001
Chromium	SA/CMC-ZFN	0.017	0.032	0.014
	commercial zeolite	0.001	0.010	0.028

According to the Thomas model, the maximum adsorption performance of copper, nickel, zinc, and chromium in industrial wastewater of SA/CMC-ZFN were determined as 0.26, 4.72, 4.96, and 0.53 mg/g, respectively, which is 9, 157, 13, and 53 times greater than commercially natural zeolite. Furthermore, the 50 percent saturation values of copper, nickel, zinc, and chromium in the column reactor filled with SA/CMC-ZFN are 3667, 4942, 6795, and 19,113 min, respectively, according to the Yoon-Nelson model, which was 4.47, 81.02, 5.08, and 43.44 times longer than zeolite. Table 9 shows the results of the breakthrough curve evaluation predicted by the equation parameter and the sum square error of each model. The experimental breakthrough curves obtained using Thomas and Yoon-Nelson's models were in good agreement with theoretical values, which were validated by their low sum of square error levels.

## Conclusions

This study, for the first time, has fabricated a low-cost and eco-friendly SA/CMC-ZFN adsorbent for the removal of Cu(II) and Cr(VI) from the aqueous solution. The characterization results revealed the successful synthesis of porous SA/CMC-ZFN and its practical application for environmental remediation. The XRD, FT-IR, SEM–EDX, and XPS analyses suggested that the possible removal mechanism of Cu(II) and Cr(VI) by SA/CMC-ZFN is governed by adsorption, reduction, precipitation, and ion exchange. The Langmuir adsorption model satisfactorily fit the isotherm adsorption equilibrium data, and the adsorption capacity of SA/CMC-ZFN for Cu(II) and Cr(VI) was 10.15 and 63.29 mg/g, respectively. The adsorption kinetics data revealed the increased removal efficiency with the increased initial concentration of the adsorbate, and adsorption followed the pseudo 2^nd^ order model, which revealed adsorption is the rate-limiting step. The process of Cu(II) and Cr(VI) removal was spontaneous, endothermic, and entropically favourable. Co-existing cations and anions did not influence Cr(VI) and Cu(II), respectively. However, SO_4_^2−^ and Pb(II) negatively impacted the removal of Cr(VI) and Cu(II), respectively.

Furthermore, SA/CMC-ZFN is inexpensive and highly efficient than that commercial natural zeolite. In the application of the continuous column process on real industrial wastewater, the experimental breakthrough curves were observed to be in good agreement with theoretical values using Thomas and Yoon-Nelson models, which were validated by their low sum square error values. Overall, the results show that SA/CMC-ZFN is an environmentally acceptable, cost-efficient, promising, and successful method for simultaneously removing heavy metals from an aqueous solution.

## Supplementary Information

Supplementary Information.
