# Supplementary material for: Portable SA/CMC entrapped bimetallic magnetic fly ash zeolite spheres for heavy metals contaminated industrial effluents treatment via batch and column studies
Source: Sci Rep. 2022 Mar 2;12:3430. doi: 10.1038/s41598-022-07274-5 (PMC8891350; doi:10.1038/s41598-022-07274-5)
Supplement: Supplementary file 1 — Supplementary Information. [file 41598_2022_7274_MOESM1_ESM.docx]

**Portable SA/CMC entrapped bimetallic magnetic fly ash zeolite spheres for heavy metals contaminated industrial effluents treatment via batch and column studies**

Ganesh Kumar Reddy Angaru^a^, Yu-Lim Choi^a^, Lakshmi Prasanna Lingamdinne^a^, Janardhan Reddy Koduru*^, a^, Jae-Kyu Yang^a^, Yoon-Young Chang**^, a^, Rama Rao Karri***^, b^

^a^Department of Environmental Engineering, Kwangwoon University, Seoul, 01897, Republic of Korea.

^b^Petroleum and Chemical Engineering, Faculty of Engineering, Universiti Teknologi Brunei, BE 1410, Brunei Darussalam.

Corresponding author’s e-mail addresses: *reddyjchem@gmail.com (J.R. Koduru); **yychang@kw.ac.kr (Y.Y. Chang); ***kramarao.iitd@gmail.com (R.R. Karri).

**Sections as per the main manuscript**

**2.1. Materials, and characteristics**

The freeze dryer was used for the synthesis of SA/CMC-ZFN, from OPERON (FDB 7003) Co., LTD. The following analysis technique was used to confirm the change in the surface properties of SA/CMC-ZFN. FT-IR (Fourier Transform Infrared Spectrometer) (Cary 610, Agilent) was used to analyze functional groups on the surface. BET (Brunauer-Emmett-Teller) specific surface area and porosity analysis of SA/CMC-ZFN was performed using ASAP 2420 (Micromeritics, USA). XRD (X-ray Diffractometer) analysis was performed using D8 Discover (Bruker AXS, USA) to observe the change in surface crystallinity of SA/CMC-ZFN. PHI Quantera-II (ULVAC-PHI, Japan) was used for XPS (X-Ray Photoelectron Spectroscopy) analysis to determine the sample surface's composition and chemical bonding state before and after adsorption. To observe the morphology of SA/CMC-ZFN, SEM (Scanning Electron Microscope, scanning electron microscope) analysis was performed using JSM-7610F (JEOL, Japan).


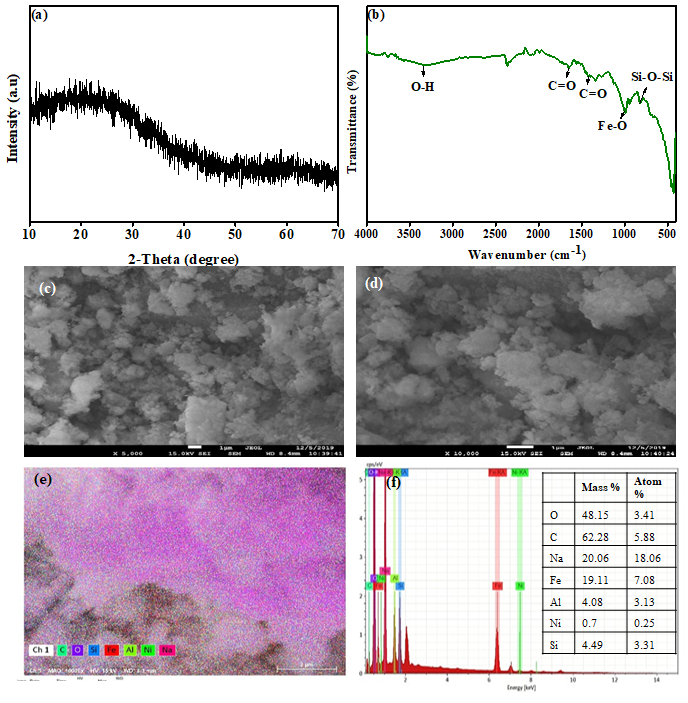


**Figure S1** : (a) X-ray diffraction pattern, and (b) Fourier transform infrared spectra of ZFN, SEM images of ZFN (c) x 5000 , (d) x 10,000; (e) EDX mapping of the element; and (f) element composition analysis.


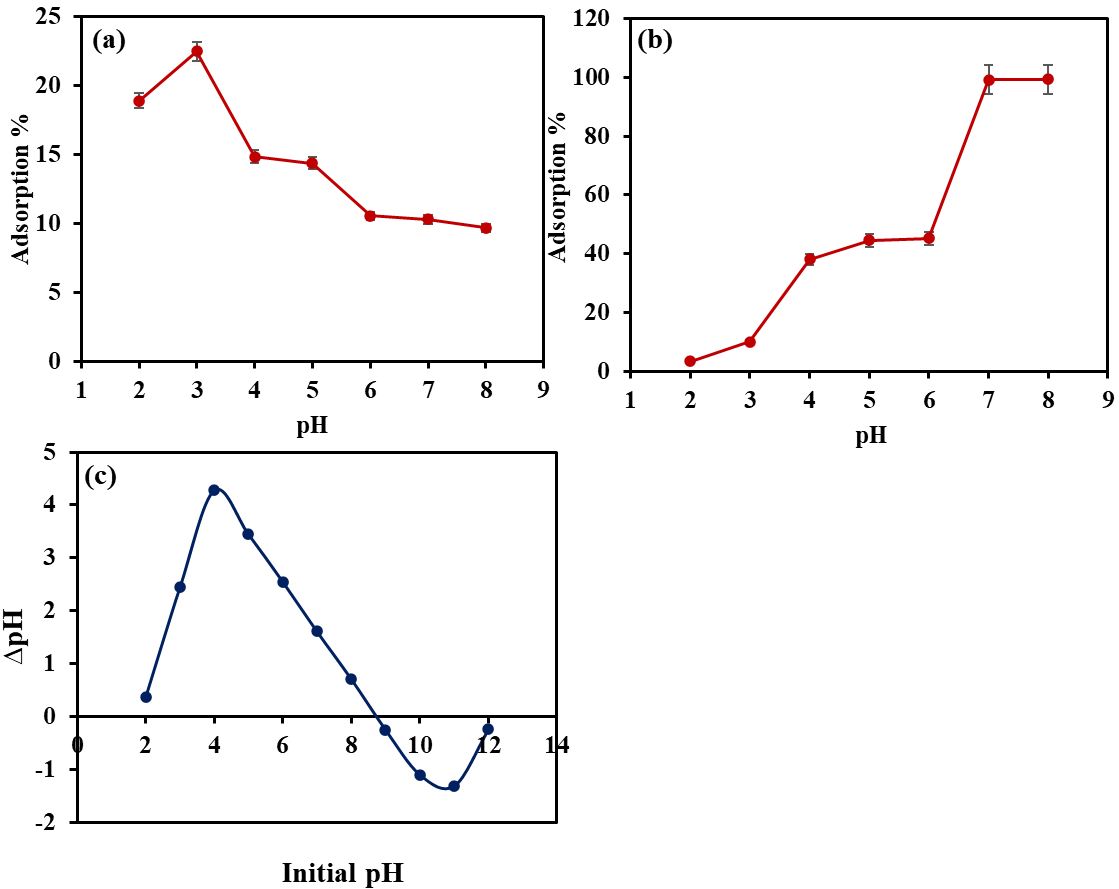


**Figure S2:** (a,b) Effect of pH on the adsorption % of SA/CMC-ZFN towards Cr(VI) and Cu(II), (c) pH_zpc_


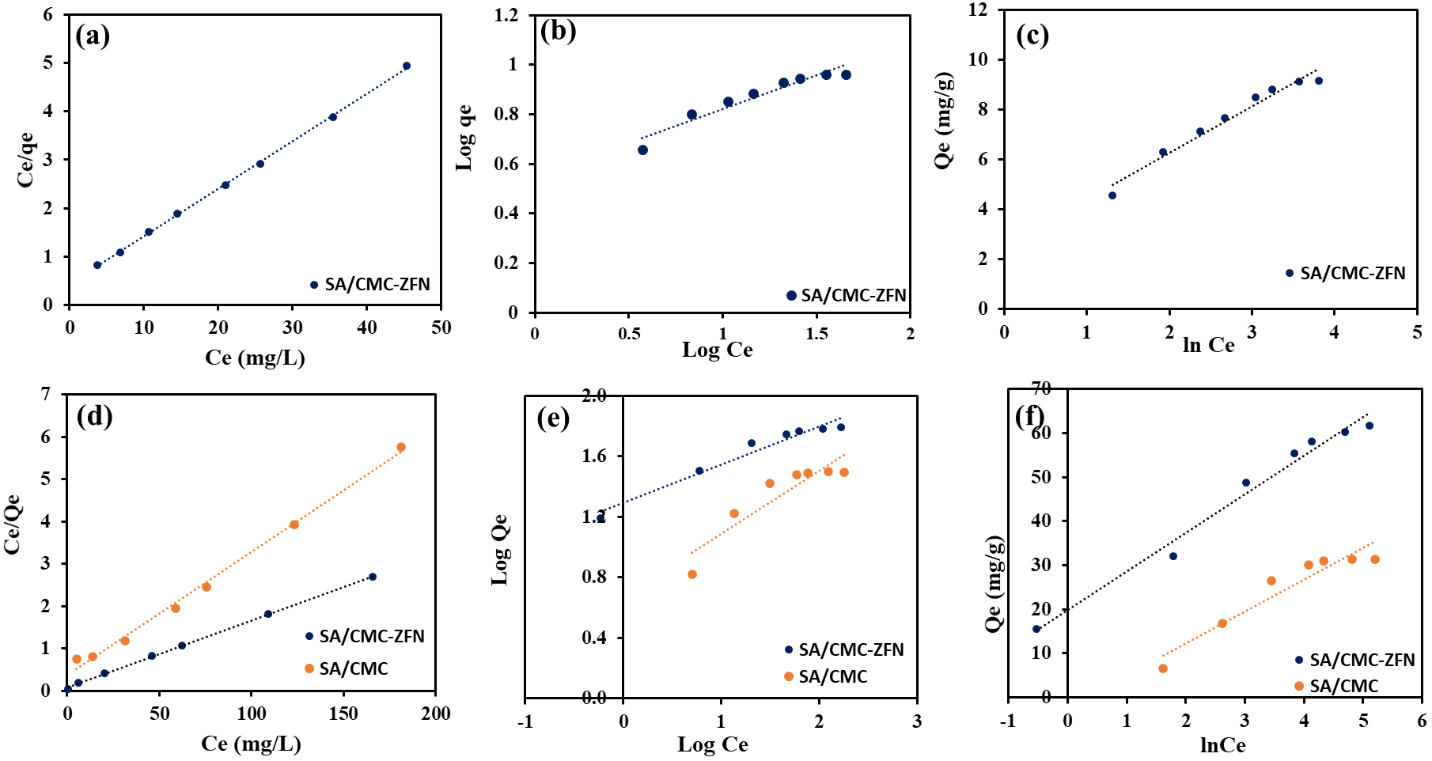


**Figure S3:** Adsorption isotherms models of SA/CMC-ZFN towards Cr(VI) and Cu(II). Langmuir model (a,d), Freundlich model (b,e), Temkin model (c,f)


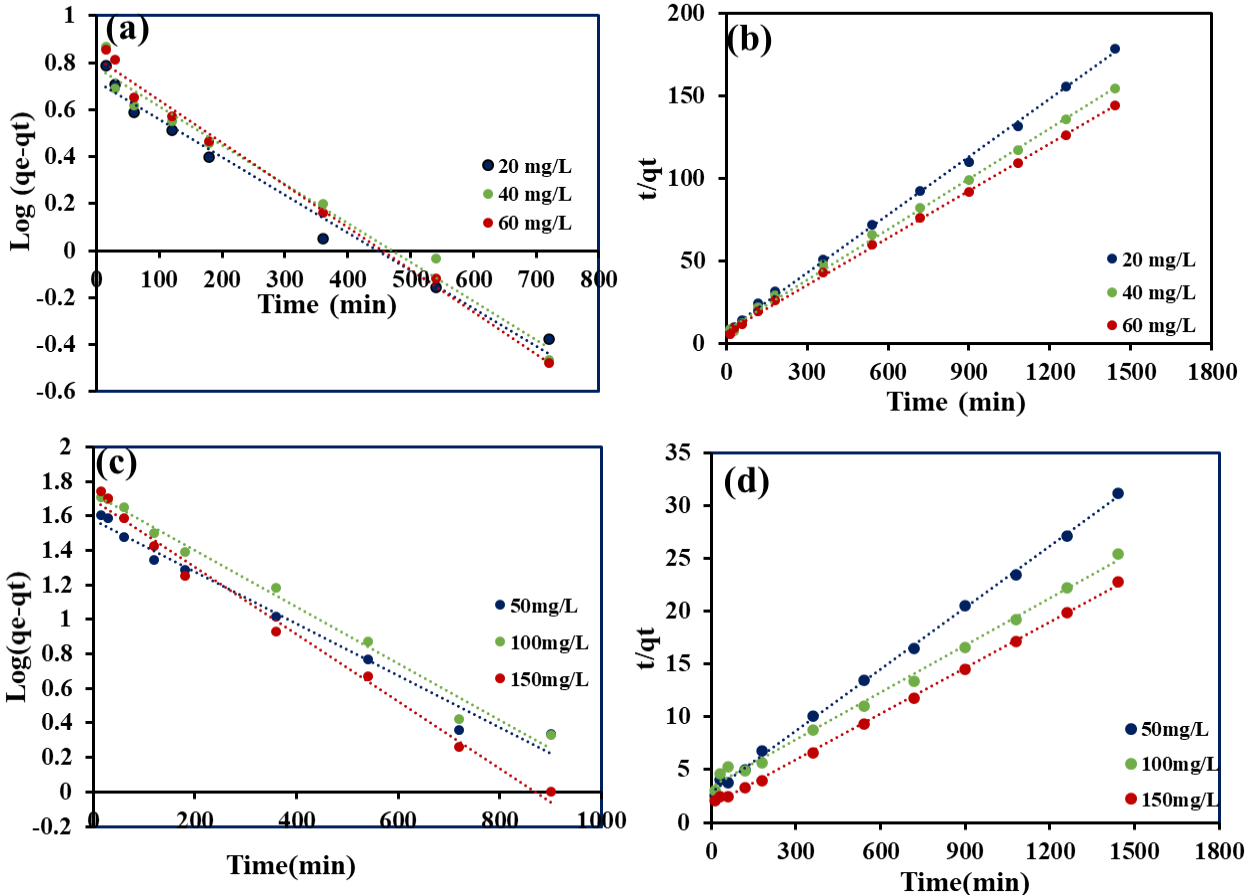


**Figure S4:** Adsorption kinetics models of SA/CMC-ZFN towards Cr(VI) and Cu(II). Pseudo first-order kinetic models (a,c), Pseudo second-order kinetic models (b,d),


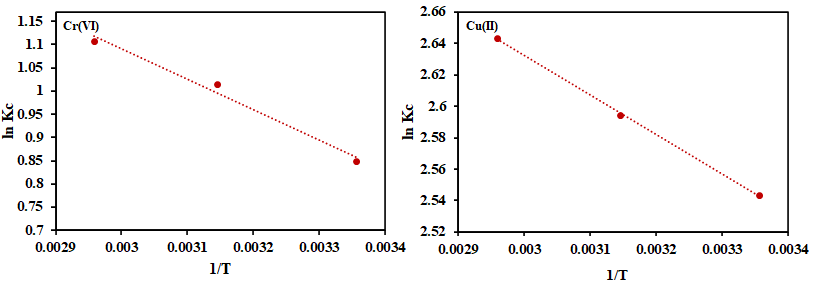


**Figure S5:** Thermodynamic studies of SA/CMC-ZFN towards Cr(VI) and Cu(II).


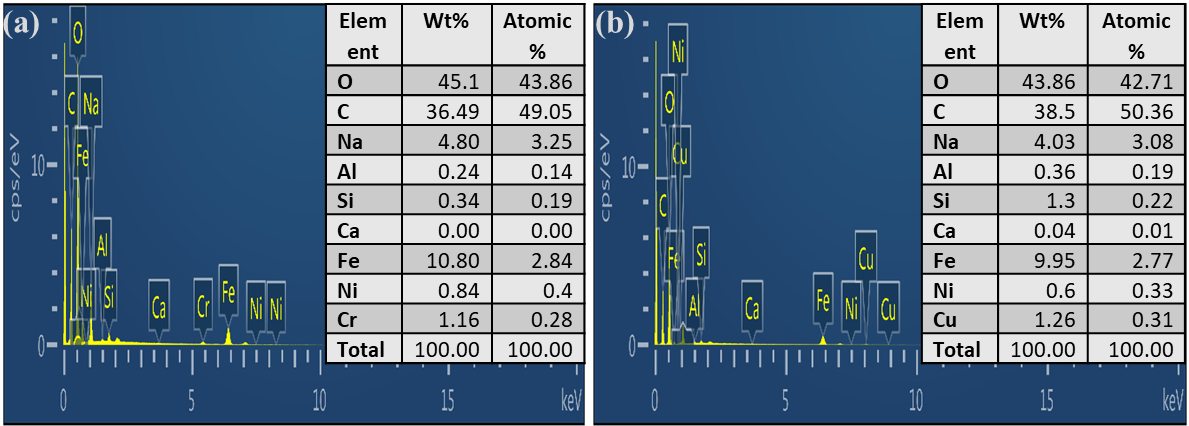


**Figure S6 :** EDX elemental composition of regenerated SA/CMC-ZFN. (a) Cr(VI)- after 3 cycles, (b) Cu(II)- after 3 cycles.
